# MEAN inhibits hepatitis C virus replication by interfering with a polypyrimidine tract‐binding protein

**DOI:** 10.1111/jcmm.12798

**Published:** 2016-03-01

**Authors:** Jihua Xue, Yanning Liu, Ying Yang, Shanshan Wu, Ying Hu, Fan Yang, Xiaotang Zhou, Jing Wang, Feng Chen, Min Zheng, Haihong Zhu, Zhi Chen

**Affiliations:** ^1^ State Key Lab of Diagnostic and Treatment of Infectious DiseasesCollaborative Innovation Center for Diagnosis and Treatment of Infectious Disease1st Affiliated Hospital of Medical SchoolZhejiang UniversityHangzhouChina

**Keywords:** anti‐HCV drug, polypyrimidine tract‐binding protein, nucleo‐cytoplasmic translocation, novel strategy

## Abstract

MEAN (6‐methoxyethylamino‐numonafide) is a small molecule compound, and here, we report that it effectively inhibits hepatitis C virus (HCV) infection in an HCV cell culture system using a JC1‐Luc chimeric virus, with a 50% effective concentration (EC50) of 2.36 ± 0.29 μM. Drug combination usage analyses demonstrated that MEAN was synergistic with interferon α, ITX5061 and ribavirin. In addition, MEAN effectively inhibits N415D mutant virus and G451R mutant viral infections. Mechanistic studies show that the treatment of HCV‐infected hepatocytes with MEAN inhibits HCV replication but not translation. Furthermore, treatment with MEAN significantly reduces polypyrimidine tract‐binding protein (PTB) levels and blocks the cytoplasmic redistribution of PTB upon infection. In the host cytoplasm, PTB is directly associated with HCV replication, and the inhibition of HCV replication by MEAN can result in the sequestration of PTB in treated nuclei. Taken together, these results indicate that MEAN is a potential therapeutic candidate for HCV infection, and the targeting of the nucleo‐cytoplasmic translocation of the host PTB protein could be a novel strategy to interrupt HCV replication.

## Introduction

Hepatitis C virus (HCV) is a leading cause of chronic hepatitis and liver‐related morbidity worldwide. Currently, the combination of pegylated interferon (IFN) and ribavirin (RBV) are no longer the standard of care because direct acting antiviral‐based regimens can now safely cure most chronic HCV infections 1. Interferon‐free and ‘all oral’ regimens are also available. However, the implementation of these approaches is complicated by the cost and the rapid emergence of resistance to antivirals. Moreover, these approaches show favourable sustained virological response only in patients who are naïve to treatment, non‐cirrhotic and non‐co‐infected with human immunodeficiency virus. Thus, new anti‐HCV therapeutic agents that are potent across different viral genotypes and cause little or no drug‐resistance induction are urgently needed.

Cell culture‐derived HCV particles, based on the Japanese fulminant hepatitis 1 (JFH1) strain of HCV genotype 2a, are infectious both *in vitro* and *in vivo* 2, 3, 4. Subsequently, to facilitate the detection of HCV infection and for the high‐throughput screening of antiviral drugs, reporter viruses have been constructed by inserting the enhanced green fluorescent protein (EGFP) or luciferase gene into the carboxyl‐terminal region of NS5A in the JFH1 and JC1 genomes 5, 6, 7. These model systems allow an assessment of the effect of antiviral agents on HCV RNA replication, virus production and infectivity and thus contribute to the rapid development of anti‐HCV agents.

Polypyrimidine tract‐binding protein (PTB), also referred to as heterogeneous nuclear ribonucleoprotein I (hnRNP I), is a 57‐kD protein that preferentially binds to the pyrimidine‐rich sequences of RNA 8. The protein is diffusely distributed throughout the nucleoplasm and is also concentrated in the perinucleolar compartment 9. Polypyrimidine tract‐binding protein consists of a nuclear export signal, nuclear localization signal and four RNA recognition motifs 10, 11. The protein can shuttle between the nucleus and cytoplasm 12, 13, a process that is tightly regulated because the diverse cellular functions of PTB are achieved by a combination of changes in its localization and its interaction with other proteins. Nuclear PTB is a negative regulator of pre‐mRNA alternative splicing, and in some cases, it regulates mRNA polyadenylation 14, 15. The shuttling of PTB plays other cellular roles, such as the transport of mature RNAs to the cytoplasm 16 and the regulation of mRNA translation and mRNA stability 17, 18, 19, 20. It has been proposed that PTB acts as an RNA chaperone, and by restructuring RNA to promote or inhibit the binding of other factors, this protein is then able to act as both a repressor and activator of RNA metabolism 8, 21, 22. The RNA chaperone activity of PTB is well documented and is known to be important for the life cycle of many viruses 23.

**Figure 1:**
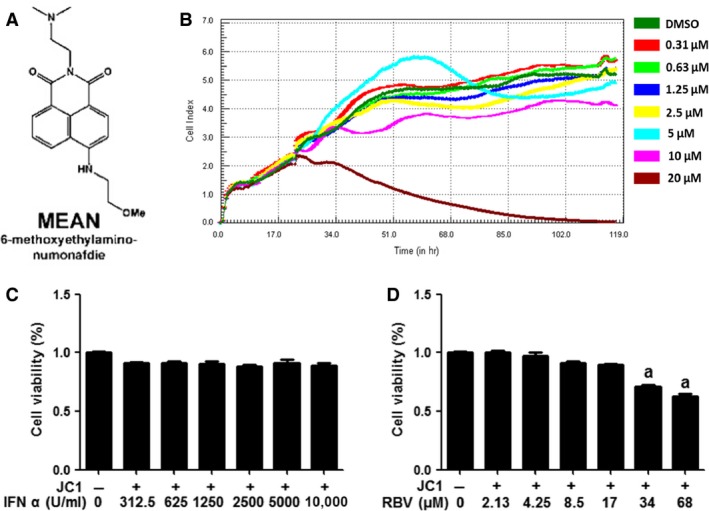
Cytotoxicity of compounds in Huh7.5.1 cells. (**A**) Chemical structure of MEAN. (**B**–**D**) Huh7.5.1 cells were grown in the presence of various concentrations of MEAN, IFN‐α or RBV for the indicated times. Cellular toxicity was determined using the RTCA method or CCK‐8 assay. The error bars represent the S.D. obtained from three independent experiments performed in triplicate. ^a^ *P* < 0.01, compared with control cells.

Amonafide is a DNA intercalator and topoisomerase II inhibitor in clinical development for the treatment of neoplastic diseases. It has a 5‐position amine that can be extensively acetylated by *N*‐acetyl‐transferase 2 to form a toxic metabolite in humans. Numonafides, 6‐amino derivatives of amonafide that avoid the toxic acetylation, also show anticancer activity 24, 25. MEAN (Fig. 1A) is a numonafide and was characterized by a gene expression profile array performed by our team, which showed its role in regulating the expression of many molecules, including PTB (data not published). Moreover, it has been reported to participate in the replication and translation of HCV. We speculated that MEAN might inhibit HCV replication by interfering with the function of PTB. To verify our hypothesis and to search for new HCV inhibitors, we examined the anti‐HCV activity of MEAN and its underlying mechanisms in an HCV cell culture system using a JC1‐Luc chimeric virus in this study.

## Materials and methods

### Compounds

The synthesis and structure‐activity of MEAN (Fig. 1A) have been previously described 24. Additional compounds tested include IFN‐α, RBV (Sigma‐Aldrich, St. Louis, MO, USA) and ITX5061. ITX5061 is an arylketoamide for which the structure has been described elsewhere.

### Cell culture and virus plasmids

Human hepatoma Huh7.5.1 cells were derived by Scott Forrest (Scripps Research Institute, La Jolla, CA, USA) and kindly provided by Dr. Jin Zhong (Institute Pasteur of Shanghai, China). Cells were grown at 37°C and 5% carbon dioxide in DMEM supplemented with 2 mM L‐glutamine, 1 mM sodium pyruvate, 1× non‐essential amino acid mix, 100 units/ml penicillin, 100 μg/ml streptomycin and 10% foetal bovine serum.

The plasmid pFL‐JC1 was a kind gift from Apath (Saint Louis, MO, USA). The chimeric full‐length construct pFL‐JC1 has been described elsewhere 3, 5, 26. The firefly luciferase gene was inserted into pFL‐JC1 to generate a plasmid pFL‐JC1‐luc for use as a viral replication reporter 27. The HCV internal ribosome entry site (IRES) dual‐luciferase reporter plasmid was a kind gift from Dr. Xinwen Chen (Chinese Academy of Sciences). This reporter plasmid was designed to indicate the level of HCV IRES‐directed translation 28.

### Cell cytotoxicity assay

The cytotoxicity of MEAN on Huh7.5.1 cells was evaluated using real‐time cellular analysis (RTCA). Briefly, the xCELLigence DP device from Roche Diagnostics (Mannheim, Germany) was used to monitor the cell cytotoxicity in real‐time. Huh7.5.1 cells (5 × 10^3^) were seeded in 96‐well electronic microtitre plates (E‐Plate^™^; Roche Diagnostics) and cultured with DMEM containing 0, 0.31, 0.63, 1.25, 2.5, 5, 10 or 20 μM of MEAN. Cell density measurements were performed in quadruplicate with a programmed signal detection every 20 min. Data acquisition and analyses were performed with the RTCA software (version 1.2; Roche Diagnostics).

The cytotoxicity of IFN‐α or RBV (Sigma‐Aldrich) was determined using the cell counting kit 8 (CCK‐8) assay (Dojindo, Kumamoto, Japan). Briefly, Huh7.5.1 cells (5 × 10^3^) were seeded into 96‐well culture plates and then treated with various concentrations of IFN‐α (0, 312.5, 625, 1250, 2500, 5000, 10,000 U/ml) or RBV (0, 2.13, 4.25, 8.5, 17, 34, 68 μM) for 48 hrs. Next, the treated cells were incubated with CCK‐8 solution (1/10 vol/vol in serum‐free media) for an additional 3 hrs at 37°C. The absorbance was determined at 450 nm using a microtitre plate reader (Bio‐Rad, Hercules, CA, USA).

### RNA transfection, HCV infection and titration

*In vitro* synthesis of HCV RNA and electroporation were performed as previously described 29. Briefly, Huh7.5.1 cells were mixed with *in vitro* transcribed RNA and electroporated (Bio‐Rad Gene Pulser System) using a single square wave at 260 V and 25‐msec. pulse length. The supernatant was harvested, and purified viruses were used for infection and titration.

### HCV infection and treatment

Huh7.5.1 cells were seeded into 96‐well plates at a density of 5 × 10^3^ cells per well in 100 μl of medium. After incubating overnight for attachment, JC1‐luc wild‐type virus, N415D mutant virus and G451R mutant virus at a multiplicity of infection of 0.01 were added to wells with or without compounds at the specified concentrations 24 hrs after infection, followed by an additional 48 hrs incubation. All conditions were run in triplicate. Hepatitis C virus infection and RNA replication rates were quantified by measuring luciferase activity using a microplate luminometer (Veritas microplate luminometer; Turner Biosystems, Sunnyvale, CA, USA). The relative light units for each condition were reported as the mean ± S.E.M. for the three wells.

### Real‐time quantitative RT‐PCR

The expression of HCV RNA was evaluated. The cells were collected by trypsinization, and total RNA was extracted using TRIzol Reagent (Invitrogen, San Diego, CA, USA) according to the manufacturer's protocol. The cDNA was produced by RT using a reverse transcription kit (Takara, Otsu, Japan). Next, the products were used for analysis on an ABI PRISM 7900 Sequence Detection System using SYBR^®^ Premix Ex Taq^™^ kit (Takara). To determine the relative quantification, the comparative threshold cycle (Ct) method was used. Each PCR assay was performed in triplicate, and the changes in mRNA levels were normalized against the levels of glyceraldehyde 3‐phosphate dehydrogenase (GAPDH). The primers purchased from Sangon Biotech (Shanghai, China) are listed in Table 2.

**Table 1:** Inhibitory activity of the compounds on wild‐type and mutant JC1‐luc virus *in vitro*

Compounds	EC50 ± S.E.M.		
	Wild‐type	N415D	G451R
MEAN (μM)	2.36 ± 0.29	3.56 ± 0.08	3.71 ± 0.14
IFN‐α (U/ml)	4.83 ± 0.57	ND	ND
RBV (μM)	EC50 > 34	ND	ND

EC50: 50% effective concentration; IFN‐α: interferon α; RBV: ribavirin; ND: not determined.

**Table 2:** Primer sequences of the nine primer sets used for RT‐PCR

Gene	Upstream primer	Downstream primer
HCV	GCGTTAGTATGAGTGTCGTG	TCGCAAGCACCCTATCAG
PTB	GGGTCGGTTCCTGCTATTCC	CGTCAGATCCCCGCTTTGTA
GAPDH	GAAGGTGAAGGTCGGAGTC	GAAGATGGTGATGGGATTTC

### Western blotting analysis

To determine the levels of HCV Core and PTB protein expression, whole‐cell extracts were prepared and fractionated using SDS‐PAGE. After electrophoresis, the proteins were electrotransferred onto nitrocellulose membranes, and blotted with monoclonal antibody specific for protein of HCV core (1:500; Abcam, Cambridge, MA, USA) or PTB (1:1000; Abcam). Blots were visualized using ECL‐associated fluorography (Pierce, Rockford, IL, USA).

### Dual‐luciferase assay

Huh7.5.1 cells (5 × 10^3^) were seeded onto 96‐well plates and transfected with 0.2 μg of the HCV IRES dual‐luciferase reporter plasmid using lipofectamine^™^ 2000 (Invitrogen). After 24 hrs of incubation, the cells were incubated with medium containing the indicated concentration of MEAN for 48 hrs. The firefly luciferase and renilla luciferase activity were measured using the Dual‐Luciferase Reporter Assay System (Promega, Madison, WI, USA) according to the manufacturer's instructions. Renilla luciferase is translated *via* cap‐dependent translation, whereas the translation of firefly luciferase is directed by the HCV IRES.

### *In vitro* combination studies

The EC50 of MEAN and the compounds was independently determined and used to establish the range of concentrations for synergy experiments. Next, compounds were tested in the following combinations: MEAN with IFN‐α, MEAN with RBV and MEAN with ITX5061. For MEAN, IFN‐α and ITX5061, the EC50 with two 2‐fold serial dilutions above and below the EC50 were selected. The ratio of the two compounds tested remained fixed across the dosing range. For RBV, which did not have a selective antiviral effect, a top dose of 17 μM was selected (below the concentration that caused the lowest cytotoxicity). Briefly, naive Huh7.5.1 cells were seeded onto 96‐well plates at a density of 5 × 10^3^ cells per well in 100 μl of DMEM culture medium. After incubating overnight for attachment, 100 μl of the JC1‐luc virus was added to the cells. After 24 hrs, the medium was aspirated, and 100 μl of serially diluted compound solutions was inoculated onto Huh7.5.1 cells in 96‐well plate. After co‐incubation for 48 hrs, Renilla luciferase expression was determined (Renilla Luciferase Assay; Promega) and reported as relative light units.

### Immunofluorescence and confocal analysis

Huh7.5.1 cells plated on glass coverslips (BD Biosciences, San Jose, CA, USA) were infected with JC1‐luc wild‐type virus for 24 hrs followed by exposure to 5 μM of MEAN for 48 hrs. Next, the ells were washed with PBS and fixed in 4% paraformaldehyde for 30 min. at room temperature (RT). After fixation, the cells were washed three times with PBS (5 min./time), permeabilized with 0.5% Triton X‐100 for 20 min., and incubated with blocking buffer (1% bovine serum albumin in PBS) for 1 hr at RT to minimize the non‐specific adsorption of the antibodies to the coverslip. The cells were incubated with primary antibodies against HCV Core (1:100; Abcam) or PTB (1:100; Abcam) overnight at 4°C. The cells were washed three times with PBS (5 min./each) and incubated with fluorescein isothiocyanate (FITC)‐conjugated secondary antibodies (1:200; Santa Cruz Biotechnology, Dallas, TX, USA) for 1 hr at RT. The nucleus was stained with 4′,6′‐diamidino‐2‐phenylindole dihydrochloride (DAPI; Sigma‐Aldrich) and the cells were washed three times (5 min./each) with PBS. After the coverslips were mounted onto glass slides with mounting medium, the glass slides were imaged using a confocal microscope (Olympus Inc., Center Valley, PA, USA). The number of core protein positive cells was counted in five randomly selected fields in each group (*n* = 3), and the mean number of infected cells was calculated for each case. In addition, the integrated optical density of each PTB‐stained Huh7.5.1 cells was determined using the Image‐Pro Plus 5.0 software (Media Cybernetics, Inc., Bethesda, MD, USA).

### RNA interference

Huh7.5.1 cells at 30–40% confluence were transfected with 100 nM PTB siRNAs (PTB1 and PTB2) synthesized by Ribobio (Guangzhou, China) with Lipofectamine^®^ RNAiMAX Reagent (Invitrogen) according to the manufacturer's protocol. Then, 24 hrs after transfection, the cells were infected with the JC1‐luc virus for 24 hrs and were collected for luciferase assay at 48 hrs post infection. In addition, non‐infected cells were also collected and lysed for real‐time quantitative RT‐PCR and Western blotting analysis at 48 hrs post transfection to determine the interfering effect of siRNAs for PTB. The sequences used here were described in previous papers 30, 31.

### Heterokaryon assays

Huh7.5.1 cells (1 × 10^5^) were transfected with 1 μg of expression plasmid encoding EGFP‐labelled PTB. At 24 hrs after transfection, these cells were co‐cultured with an equal number of murine NIH 3T3 cells for 2 hrs and then further incubated for an additional 2 hrs in the presence of 100 μg/ml cycloheximide (Sigma‐Aldrich). Cell fusions were performed by washing the cells with PBS, incubating them in 50% (wt/vol) polyethyleneglycol 1500 (Roche Molecular Biochemicals, Basel, Switzerland) for 2 min., and rinsing them with PBS. Heterokaryons were further incubated in media containing 100 μg/ml cycloheximide and 5 μM MEAN for 5 hrs. The cells were stained with DAPI (200 ng/ml) (Sigma‐Aldrich) for 1 min. to identify heterokaryons.

### Data analysis

All data were processed using SPSS 19.0 software (SPSS Inc.,Chicago, IL, USA) and are presented as the mean ± S.E. anova and fisher's least significant difference (LSD) test were used for comparisons among groups and between paired data respectively. When the data were not normally distributed, the Mann–Whitney *U*‐test and a one‐way non‐parametric anova (Kruskal–Wallis test) were performed to compare quantitative variables between two groups of observation and in more than two groups of data respectively. A *P*‐value less than 0.05 was considered significant.

Determinations of compound interactions were quantified on the basis of the median‐effect principle. Combination indices (CIs) were determined using CalcuSyn (Biosoft, Great Shelford, Cambridge, UK) for each experiment at EC50, 75% effective concentration (EC75), and 90% effective concentration (EC90) levels. Five replicates per condition were evaluated. A CI <0.9 was considered synergistic, CI ≥0.9 and ≤1.1 was considered additive and a CI >1.1 was deemed antagonistic.

## Results

### Cytotoxicity of compounds

Prior to the analysis of the potential antiviral effect of MEAN on HCV, we first determined its toxicity on Huh7.5.1 cells. Although some evidence of toxicity was observed at 20 μM, a concentration of 10 μM was shown to have no toxic effect (Fig. 1B). The cytotoxicity of IFN‐α and RBV in Huh7.5.1 was also evaluated (Fig. 1C and D). No cytotoxicity was detected, even when the cells were treated with 10,000 U/ml of IFN‐α. However, some evidence of toxicity was found when the cells were exposed to RBV at a concentration of 34 μM.

### MEAN inhibits JC1‐luc wild‐type virus infection

**Figure 2:**
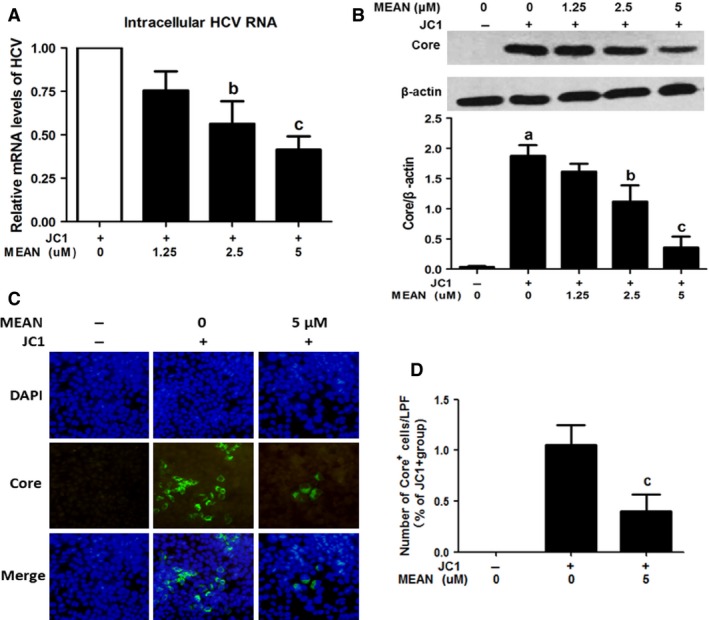
Effects of MEAN on HCV RNA synthesis and protein expression. Huh7.5.1 cells were infected with the JC1 virus for 24 hrs and then grown in the presence of the indicated concentrations of MEAN. Naive Huh7.5.1 cells were used as a mock‐infected control. (**A**) The intracellular HCV RNA level was determined using real‐time quantitative RT‐PCR and normalized against the cellular GAPDH level at 72 hrs post infection. These results are expressed as the mean ± S.D. from three independent experiments performed in triplicate. The data are expressed as the per cent inhibition relative to the control. (**B**) Intracellular HCV Core protein levels were analysed using Western blotting analysis at 72 hrs post infection and their intensities were quantified using Quantity One v4.62 and normalized against the internal reference control β‐actin. The results of three independent experiments are expressed as the mean ± S.D. (**C**) Imaging of HCV core protein fluorescence intensity. Representative images of three independent experiments are presented. (**D**) Quantifications of core^+^ cells in each group. ^a^ *P* < 0.01, compared with control cells; ^b^ *P* < 0.05, compared with JC1‐infected cells; ^c^ *P* < 0.01, compared with JC1‐infected cells.

Huh7.5.1 cells infected with the JC1‐luc virus were incubated with increasing doses of MEAN. Hepatitis C virus infection and replication efficiency were analysed using the luciferase assay. These results indicated that MEAN was a potent inhibitor of JC1‐Luc replication, with an EC50 of 2.36 ± 0.29 μM (Table 1). The anti‐HCV activity of MEAN in Huh7.5.1 cells infected with the JC1‐luc virus was also examined at the RNA and protein levels using real‐time quantitative RT‐PCR and Western blotting analysis respectively. These results demonstrated that both HCV RNA and core protein expression levels were markedly reduced when the cells were infected with the JC1‐luc virus in the presence of MEAN at 24 hrs post infection (*P* < 0.05 or *P* < 0.01; Fig. 2A and B). The inhibitory effect of MEAN on HCV replication was also validated using an immunofluorescence assay. Figure 2C and D showed that MEAN treatment could reduce the number of core^+^ cells found in JC1‐luc virus‐infected cells (*P* < 0.01). These results suggest that MEAN significantly inhibited HCV infection. The inhibitory activity on HCV replication of IFN‐α and RBV was also assessed. The EC50 values for MEAN and each of the companion compounds studied in the JC1‐luc assay system are listed in Table 1.

### MEAN inhibits N415D mutant virus and G451R mutant virus infections

**Figure 3:**
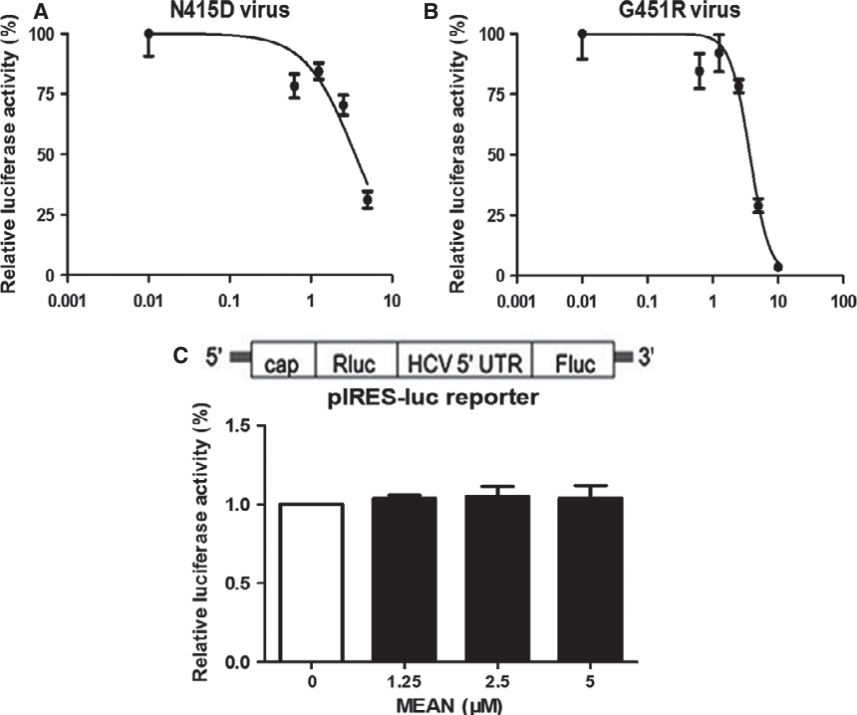
Effect of MEAN on N415D mutant virus and G451R mutant virus infections and HCV IRES‐mediated translation. (**A** and **B**) Huh7.5.1 cells were infected with N415D virus or G451R virus for 24 hrs and then overlaid with fresh medium containing increasing concentrations of MEAN. At 3 days after infection, the cells were lysed and measured using a luciferase assay. Representative inhibition curves are plotted. Concentrations of MEAN are indicated on the *x*‐axis, and the measured luminescent signals are expressed as a percentage of signal obtained in no‐treatment controls on the *y*‐axis. Symbols indicate the mean ± S.D. for triplicate determinations in a single experiment. (**C**) Huh7.5.1 cells were transiently transfected with the dual‐luciferase reporter gene construct containing HCV IRES elements (5′ UTR) and then treated with increasing doses of MEAN. Luciferase assays were performed at 72 hrs post transfection. The relative luciferase activity was expressed as the ratio of firefly luciferase activity to renilla luciferase activity. The results are expressed as the mean ± S.D. from three independent experiments performed in triplicate.

The effect of MEAN on N415D mutant virus and G451R mutant virus infections was explored using a luciferase assay. Figure 3A and B showed that MEAN could inhibit N415D mutant virus and G451R mutant virus infections, and the EC50s were 3.56 ± 0.08 μM and 3.71 ± 0.14 μM respectively (Table 1).

### MEAN fails to down‐regulate HCV IRES‐directed translation

The effect of MEAN on HCV IRES‐directed translation was further investigated. Transient transfection assays were performed with a dual‐luciferase reporter gene construct containing the HCV IRES element 32. This reporter plasmid was designed to indicate the level of HCV IRES‐directed translation, in which the translation of the upstream renilla luciferase gene was mediated by the 5′cap structure, and the downstream firefly luciferase gene was controlled by an HCV IRES element (Fig. 3C). As shown in Figure 3C, no significant differences were observed during treatment with increasing amounts of MEAN, suggesting that MEAN had no significant effect on HCV IRES‐directed translation.

### MEAN is synergistic with IFN‐α, RBV and ITX5061

**Figure 4:**
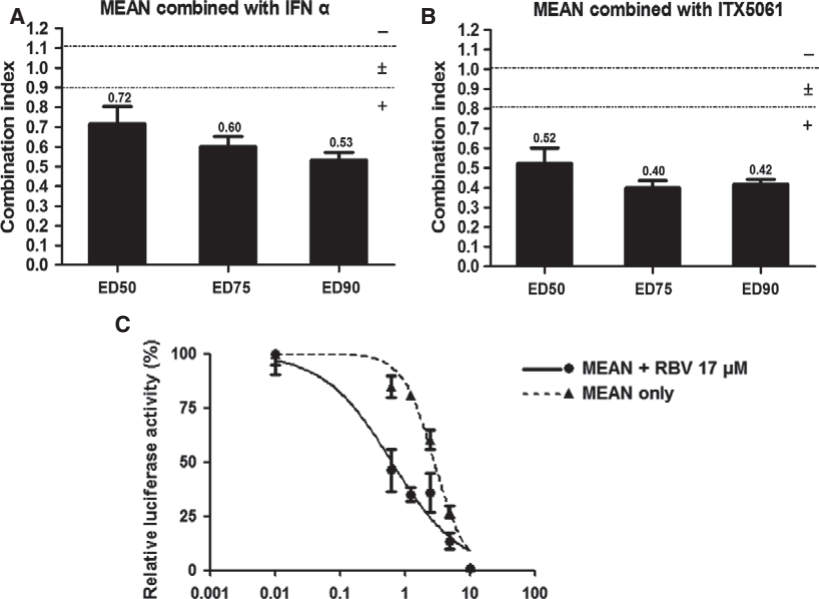
Combinatorial treatment of MEAN with various anti‐HCV compounds. Huh7.5.1 cells were infected with the JC1 virus for 24 hrs and then grown in the presence of the indicated concentrations of compounds. (**A** and **B**) Combination index (CI) of MEAN in combination with IFN‐α or ITX 5061. Numerical values above the bars indicate the mean CI. Error bars represent the S.E.M. of the CI. The two lines at 0.9 and 1.1 represent the bounds of an additive interaction. +, synergy; ±, additivity; ‐, antagonism. The 50% effective dose (ED50), ED75 and ED90 refer to the combination index at the 50% effective concentration (EC50), EC75 and EC90, respectively, of each compound. (**C**) Combination of MEAN with RBV. JC1 virus‐infected Huh7.5.1 cells were treated with various concentrations of MEAN in combination RBV. Viral replication was measured using the luciferase assay 72 hrs after infection.

Figure 4A and B show that MEAN was synergistic with IFN‐α and ITX5061 at the 50% effective dose (ED50), ED75 and ED90. Although RBV alone did not exhibit strong antiviral activities *in vitro*, it is a key component of the standard of care in patients. Varying amounts of MEAN were co‐incubated with a high concentration of RBV (17 μM), and the data revealed that MEAN had moderate antiviral synergy when combined with RBV (Fig. 4C).

### Knockdown of PTB inhibits HCV infection using JC1‐luc virus

**Figure 5:**
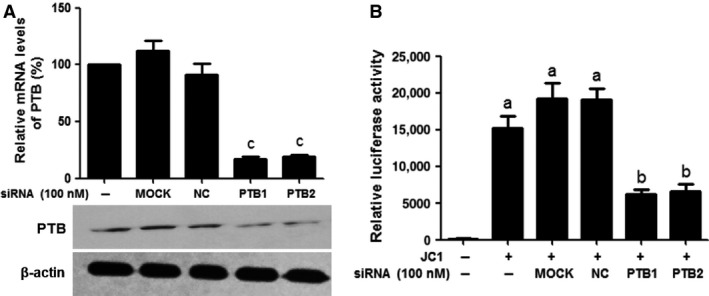
Effects of siRNAs for PTB on the replication of HCV. (**A**) Silencing effects of siRNAs on the mRNA and protein expression levels of PTB. Huh7.5.1 cells were treated with siRNAs specific to PTB (PTB1 and PTB2), an irrelevant siRNA (NC) was used as control in each experiment. GAPDH mRNA and β‐actin protein were used for normalization. The results represent three independent experiments. (**B**) Inhibition of the replication of HCV using the luciferase assay. Naive Huh7.5.1 cells were used as a mock‐infected control. Data represent the mean ± S.D. of three independent experiments. ^a^ *P* < 0.01, compared with control cells; ^b^ *P* < 0.05, compared with JC1‐infected cells; ^c^ *P* < 0.01, compared with MOCK‐treated cells.

The silencing effects of siRNAs on the mRNA and protein expression levels of PTB were evaluated using real‐time quantitative RT‐PCR and Western blotting analyses. Figure 5A shows that compared with the MOCK‐treated group, PTB mRNA and protein levels in PTB siRNA‐treated cells were significantly decreased (*P* < 0.01). Inhibition of the replication of HCV was measured using a luciferase assay. Figure 5B shows that the replication of HCV was also dramatically inhibited by PTB1 and PTB2 (*P* < 0.05).

### MEAN down‐regulates mRNA and protein expression levels of PTB

**Figure 6:**
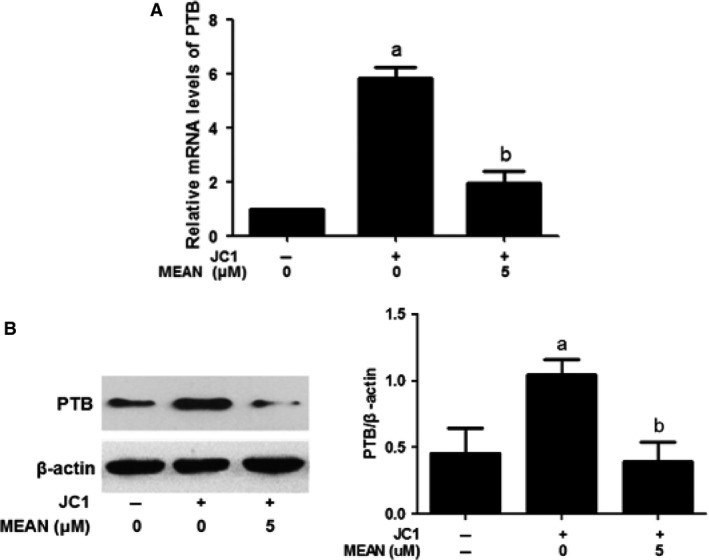
Effects of MEAN on PTB RNA synthesis and protein expression. Huh7.5.1 cells were infected with the JC1 virus for 24 hrs and then grown in the presence of the indicated concentration of MEAN. Naive Huh7.5.1 cells were used as a mock‐infected control. (**A**) The intracellular HCV RNA level was determined using real‐time quantitative RT‐PCR and normalized against cellular GAPDH level at 72 hrs post infection. The results are expressed as the mean ± S.D. from three independent experiments performed in triplicate. (**B**) The intracellular PTB protein level was analysed using Western blotting analysis at 72 hrs post infection. β‐actin was used as internal reference controls. ^a^ *P* < 0.01, compared with control cells; ^b^ *P* < 0.05, compared with JC1‐infected cells.

The expression of PTB was assessed using real‐time quantitative RT‐PCR and Western blotting analyses. These results indicate that both PTB RNA and protein expression levels induced by JC1 virus infection were markedly reduced when the cells were treated with 5 μM MEAN at 24 hrs post infection (*P* < 0.01; Fig. 6A and B).

### MEAN reduces the cytoplasmic translocation of PTB

**Figure 7:**
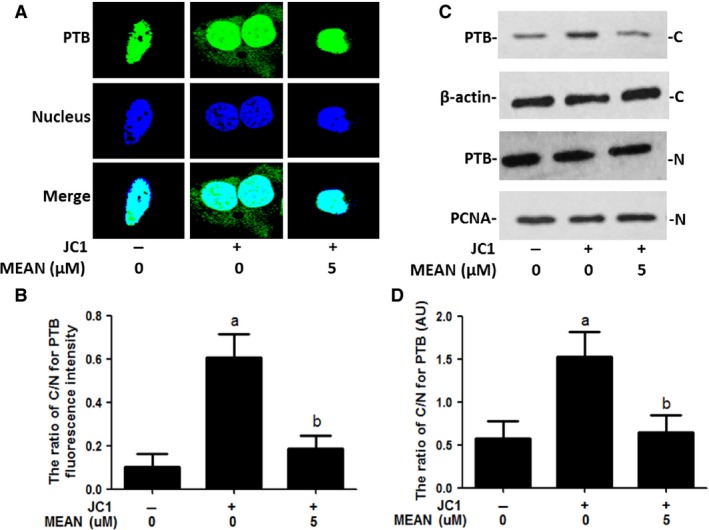
Effect of MEAN on nucleo‐cytoplasmic shuttling of PTB. Huh7.5.1 cells were infected with the JC1 virus and then grown in the presence of 5 μM of MEAN for 48 hrs. Naive Huh7.5.1 cells were used as a mock‐infected control. (**A**) Imaging of PTB fluorescence intensity. Cellular PTB was stained with anti‐PTB mouse antibody and then stained with Alexa Fluor 488 anti‐rabbit secondary antibody (green colour). The nucleus was stained with DAPI (blue colour). Merge indicates the combination of PTB fluorescence intensity with nuclear fluorescence intensity. (**B**) The ratio of cytoplasm to nuclear PTB fluorescence intensity. Data of three independent experiments represent the mean ± S.D. (*n* = 10). (**C** and **D**) The cytoplasm to nuclear ratio for PTB expression levels using a Western blotting assay. C: cytoplasm; N: nucleus; PCNA: proliferating cell nuclear Ag. The representative blots of three independent experiments are shown. ^a^ *P* < 0.01, compared with control cells; ^b^ *P* < 0.05, compared with JC1‐infected cells.

**Figure 8:**
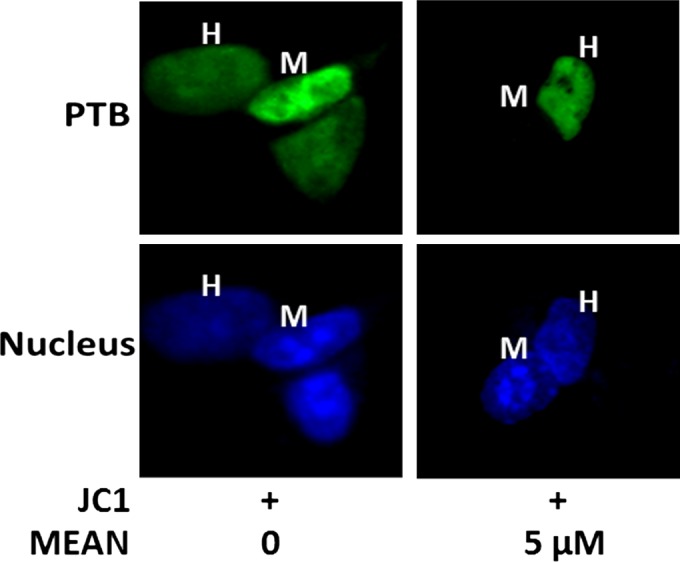
Nucleo‐cytoplasmic shuttling of EGFP‐PTB is inhibited by MEAN as validated in human‐mouse heterokaryons. EGFP‐PTB transfected or non‐transfected Huh 7.5.1 cells were fused with NIH 3T3 cells to form heterokaryons and were incubated in the presence of cycloheximide to prevent new protein synthesis. Cells were fixed 5 hrs after heterokaryon formation, and the localization of GFP‐PTB was visualized using fluorescence microscopy. Human and mouse cell nuclei (H and M respectively) were distinguished by staining the cells with the DNA dye DAPI, which reveals characteristic dense DNA clusters in the mouse nuclei but not in human nuclei.

The cytoplasmic translocation of PTB is a key process in virus infection. Western blotting analyses, immunofluorescence analysis and heterokaryon assays were performed to examine the effect of MEAN on virus‐induced PTB cytoplasmic accumulation. The results of the Western blotting analyses showed that PTB was predominantly localized in the nucleus in the absence of HCV infection; but strong cytoplasmic accumulation of PTB was observed upon HCV stimulation. Treatment by MEAN significantly blocked the cytoplasmic redistribution of PTB upon infection (*P* < 0.05; Fig. 7C and D). The results of the immunofluorescence assay also showed that the ratio of nuclear to cytoplasm PTB according to fluorescence intensity in MEAN‐treated cells was significantly lower compared to HCV‐infected cells (*P* < 0.05; Fig. 7A and B). In addition, a heterokaryon assay was performed to confirm the sequestered effect of MEAN on the nucleo‐cytoplasmic shuttling of PTB. When EGFP‐PTB‐transfected Huh7.5.1 cells were fused with NIH 3T3 cells in the absence of protein synthesis, EGFP‐PTB was detected in the mouse nuclei of the heterokaryons, indicating that EGFP‐PTB was exported from the human nuclei and reimported into the mouse nuclei, but the redistribution of EGFP‐PTB could be blocked by MEAN treatment (Fig. 8).

## Discussion

Hepatitis C virus is dependent on host cells to provide numerous factors for its replication. The goal of our study is to learn whether we can suppress viral replication by regulating the host cell environment rather than targeting viral enzymes. Using this strategy, the chemotherapeutic pressure is not on viral components; thus, antiviral agents working through this mechanism might inhibit viral replication with little to no induction of drug‐resistant mutations.

Using the reporter replicon, we discovered that the small molecule compound MEAN is a potent inhibitor of HCV replication, and the result was confirmed at the RNA and protein levels by detecting HCV mRNA and HCV core proteins using real‐time quantitative RT‐PCR and Western blotting analyses respectively. In addition, the results of the immunofluorescence assay showed that MEAN treatment could reduce the number of core^+^ cells found in JC1‐luc virus‐infected cells. *In vitro* combinatorial experiments indicated that MEAN demonstrated synergistic antiviral activity when combined with the standard of care agents, IFN‐α and RBV or ITX5061.

As a ubiquitous RNA‐binding protein, PTB is primarily localized in the nucleus, where its main function is to regulate the alternative splicing of many pre‐mRNAs 10, 33. However, the protein can be shuttled from the nucleus to the cytoplasm in response to specific signals, such as viral infection 34. An effect of PTB on several different cytoplasmic events, such as mRNA localization, mRNA translation and mRNA stability, has been reported 16, 17, 18, 19, 20. Moreover, an extensively studied role of cytoplasmic PTB is in the regulation of IRES‐dependent translation of several cellular and viral mRNAs.

A role for PTB in HCV replication and HCV IRES‐mediated translation has been proposed. Aizaki *et al*. showed that PTB was a component of the HCV RNA replication complex and is necessary for RNA synthesis 35. Chang and Luo reported that PTB was required for efficient replication of HCV RNA 36. Other studies have also suggested that recognition of the 3′non‐translated region (NTR) by PTB was involved in the initiation and/or regulation of HCV RNA replication 37, 38, 39. Several studies also indicated that the interaction of PTB with the 5′NTR and 3′NTR of the HCV RNA is required for the initiation of translation 40, 41.

The necessity of PTB for HCV replication in these above studies was demonstrated using cells harbouring an HCV replicon but not authentic cell culture‐derived viruses. Thus, the role of PTB in HCV replication was first confirmed in an HCV cell culture system using a JC1‐Luc chimeric virus in our study. Furthermore, the results indicated that knockdown of PTB expression with siRNAs significantly reduced HCV replication. We considered that PTB might be a target against HCV and our study translated their achievement into drug discovery.

Increased expression and nucleo‐cytoplasmic translocation of PTB in response to HCV infection were observed in our study. However, treatment of HCV‐infected hepatocytes with MEAN significantly reduced the PTB levels and blocked the cytoplasmic redistribution of PTB upon infection. Because PTB in the host cytoplasm is directly associated with HCV replication, inhibition of HCV replication by MEAN can result in the sequestration of PTB in treated nuclei.

Although MEAN blocks the cytoplasmic redistribution of PTB, it has no inhibitory effect on HCV IRES‐directed translation according to the results of the dual‐luciferase assay. The reason may be that PTB‐5′IRES binding was much weaker than PTB‐3′NTR binding 39, 42. In addition, strong, selective and preferential binding of PTB to the 3′end of the HCV genome suggests that it may be recruited to participate in viral replication, facilitating the direct initiation of negative strand RNA synthesis, stabilizing the viral genome, and/or regulating the encapsidation of genomic RNA, but it is not involved in viral translation.

In summary, our results demonstrated that the small molecule compound MEAN could inhibit HCV replication by down‐regulating the nucleo‐cytoplasmic translocation of PTB. Using host PTB as a new target to control HCV infection represents a novel strategy against HCV. Its mode of action is distinctly different from that of the anti‐HCV drugs currently used in clinic, NS3/4A inhibitors and NS5A/NS5B inhibitors. Because this target is not a viral enzyme, antiviral agents acting through this mechanism might inhibit viral infection with no or a decreased chance of causing drug‐resistant mutations. Ideally, a combination of host and viral inhibitors will provide a variety of drug regimens that are appropriate for different patients. We propose that the combination of a PTB suppressor with known anti‐HCV drugs might provide a new regimen for improved therapeutic efficacy in the treatment of hepatitis C and may provide the potential advantage of preventing or decreasing drug‐resistant mutations in virus.

## Conflicts of interest

The authors confirm that there are no conflicts of interest.

## Supporting information


**Figure S1** Effect of MEAN at various concentrations on HCV replication.
