# Supplementary figures and images for: MEAN inhibits hepatitis C virus replication by interfering with a polypyrimidine tract‐binding protein
Source: J Cell Mol Med. 2016 Mar 1;20(7):1255–65. doi: 10.1111/jcmm.12798 (PMC4929307; doi:10.1111/jcmm.12798)

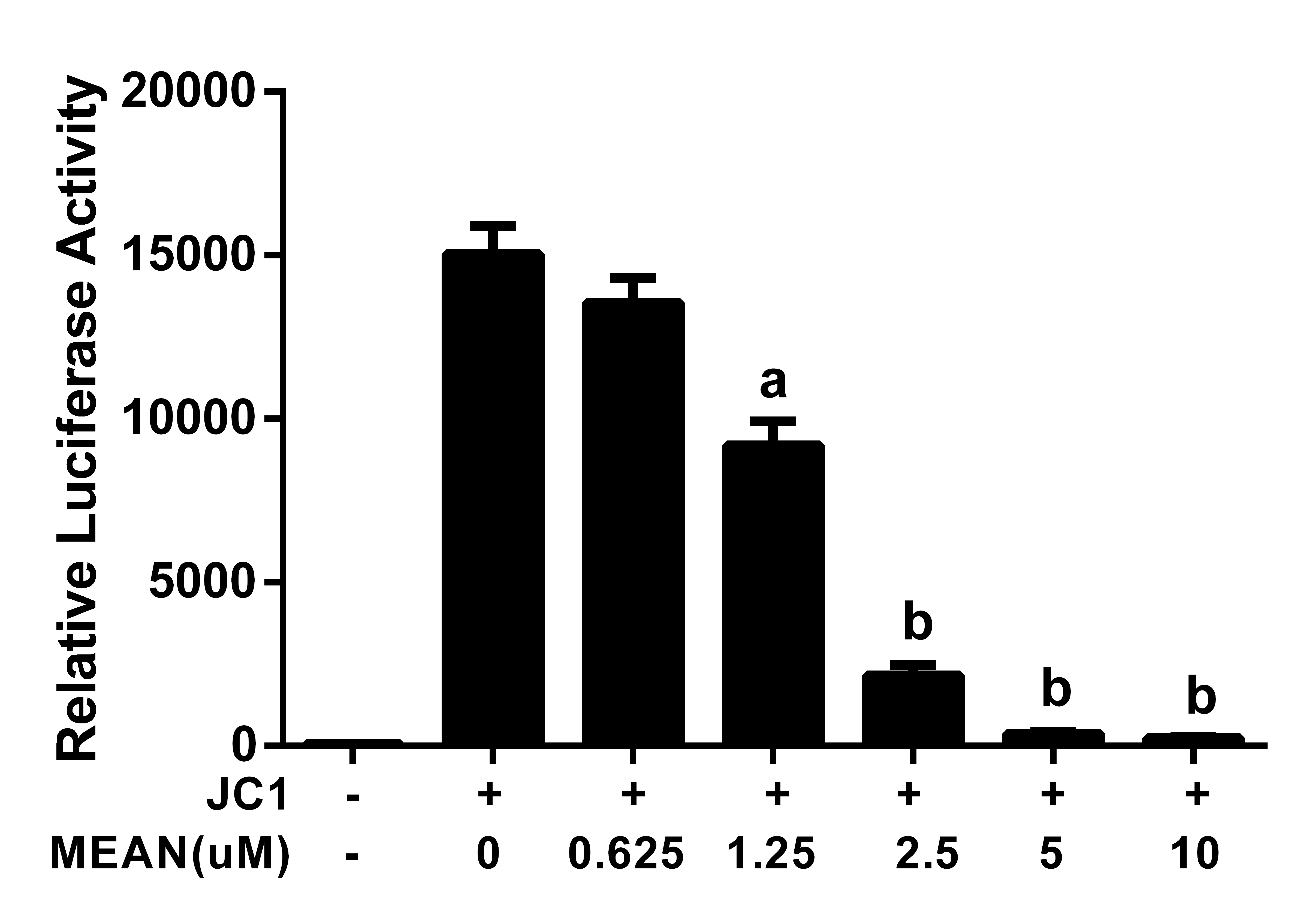

Supplement: Supplementary file 1 — Figure S1 Effect of MEAN at various concentrations on HCV replication. [file JCMM-20-1255-s001.tiff]
